# Identification of Novel Microsatellite Markers to Assess the Population Structure and Genetic Differentiation of *Ustilago hordei* Causing Covered Smut of Barley

**DOI:** 10.3389/fmicb.2019.02929

**Published:** 2020-01-15

**Authors:** Prem Lal Kashyap, Sudheer Kumar, Ravi Shekhar Kumar, Rahul Tripathi, Palika Sharma, Anju Sharma, Poonam Jasrotia, Gyanendra Pratap Singh

**Affiliations:** ICAR – Indian Institute of Wheat and Barley Research, Karnal, India

**Keywords:** barley, covered smut, genetic diversity, gene flow, genome, haplotype, microsatellite, population structure

## Abstract

Barley covered smut (CS) pathogen *Ustilago hordei* genome was mined for microsatellite distribution and their application in defining population structure and genetic variation. To dissect the molecular variation and genetic structure of *U. hordei*, 59 fungal isolates representing two distinct agro-ecological zones of India were analyzed by employing simple sequence repeats (SSRs). Using bioinformatic approaches, a total of 100,239 and 137,442 microsatellites were identified from 20.13 and 26.94 Mb of assembled genomic sequences of Uh364 and Uh4857-4 isolates of *U. hordei*, respectively. Penta-nucleotides (31.29 and 29.75%) followed by tri-nucleotide (28.27 and 29.88%) were most prevalent in both the genomes. Out of them, 15 polymorphic microsatellites showing conservancies in both the genomes were selected for exploring population genetic structure of *U. hordei*. An average of two alleles per microsatellite marker was generated with band size ranging from 180 to 850 bp. Polymorphic information content (PIC) varied between 0.095 and 0.37. Fifty-nine isolates were distributed in two distinct groups with about 65% genetic similarity according to UPGMA clustering and population structure analysis (*K* = 2). Gene flow analysis (Nm = 1.009) reflected moderate gene flow among the analyzed population. An analysis of molecular variance (AMOVA) displayed high level of genetic variation within population (87%) and low variation among populations (13%). Linkage disequilibrium (LD) analysis indicated positively significant but relatively low standardized index of association (SIA) value in both the population sets (SIA = 0.181), advocating a state of LD with epidemic population structure. In conclusion, the newly developed neutral SSR markers are highly polymorphic within *U. hordei* and will be useful for revealing evolutionary history and providing deep insight into the population dynamics of *U. hordei* in India as well as facilitating developing management strategies for CS of barley.

## Introduction

Covered smut (CS) incited by biotrophic fungus, *Ustilago hordei*, is one of the most important diseases of barley (*Hordeum vulgare* L.). It is a seed- and soil-borne disease and fungal inocula survive in the form of teliospores on the external surface of seeds and in the soil (Grewal et al., 2008). Usually, dikaryotic mycelia attack the plant at seed germination stage via coleoptiles and move toward meristematic region of shoot with the growth of barley seedlings (Kozar, 1969; Hu et al., 2002). At the flowering stage, the mycelia penetrate in the inflorescence and produce diploid teliospores in place of kernels (Grewal et al., 2008). The typical symptom produced by CS is the black mass of spores in the infected barley head covered with a persistent membrane (Thomas, 1988). The membrane burst during harvesting and threshing, discharging teliospores that contaminate healthy seeds or drop down on the soil surface (Mathre, 1997). CS disease can be successfully managed by using disease-free seed, recommended seed treatment with fungicides, and resistant cultivars (Grewal et al., 2008). Seed treatment with Carbendazim, Tebuconazole, and Carboxin is effective (Kaur et al., 2014). However, farmers incur additional costs for chemicals and are not an option for organic production system. Moreover, pathogen may develop resistance (Ben-yephet et al., 1975; Henry et al., 1987), intimidating to make chemical seed treatment futile. Under such circumstances, understanding the virulence of the pathogen and population genetic structure of *U. hordei* would be helpful to devise better management strategies for this important fungal pathogen.

Traditionally, variations among fungal pathogens have been studied by analyzing the cultural characteristics, morphology, sporulation, virulence, mating type, plant–fungus physiological and biochemical interactions, and disease response on differential hosts (Rau et al., 2005; Kashyap et al., 2016; Yu et al., 2016; Goswami et al., 2019). Unfortunately, these techniques are laborious and time-consuming and also influenced by the environment. Moreover, they are not very accurate and precise. However, DNA-based markers have the potential to decipher genetic variation of fungal pathogens and are documented as precise and accurate (Weising et al., 1995; Kumar et al., 2013; Kashyap et al., 2015; Prasad et al., 2018). Several types of molecular markers such as random amplified polymorphic DNA (RAPD), amplified fragment length polymorphism (AFLP), inter-simple sequence repeats (ISSR), and single-nucleotide polymorphism (SNP) have been widely explored for understanding the genetic diversity and population structure of fungal crop pathogens (Karwasra et al., 2002; Braithwaite et al., 2004; Sun et al., 2013; Goswami et al., 2017). However, in comparison to these markers, SSR emerged as a promising marker for DNA fingerprinting, genetic linkage mapping, quantitative trait locus (QTL) identification, population genetics, and evolutionary studies owing to their polymorphic attribute, excellent reproducibility, co-dominance, and omnipresence inside the genome (Ellegren, 2004; Kumar et al., 2012; Singh et al., 2014a; Rai et al., 2016). Recently, these markers have been explored for studying various phytopathogenic fungi, such as *Ustilago segetum tritici* (Kashyap et al., 2019), *Tilletia indica* (Sharma et al., 2018), *Ustilago scitaminea* (Zhou et al., 2008), *Ustilago maydis* (Jiménez-Becerril et al., 2018), *Phytophthora infestans* (Montarry et al., 2010), *Fusarium udum* (Kashyap et al., 2015), *Fusarium pseudograminearum* (Scott and Chakraborty, 2010), and *Colletotrichum gloeosporioides* (Moges et al., 2016). So far, a single report is available with a limited number of SSR markers for the analysis of genetic variation and population structure of *U. hordei* (Yu et al., 2016) and no comprehensive database of such markers are documented through complete genome-wide SSR mining. At present, draft genome of two isolates of *U. hordei* (Uh4857-4 and Uh364) is available in National Center for Biotechnology Information (NCBI) data bank^1^. Therefore, in the present study, attempts have been made to explore this genomic resource for identification, characterization, and development of microsatellites markers. Further, efforts have been put to characterize *U. hordei* isolates collected from diverse barley-growing regions of North India by validating polymorphic SSR markers and to identify various factors that determine *U. hordei* population structure.

## Materials and Methods

### Sequence Resources and Bioinformatics Analysis

The whole genome sequences of two *U. hordei* isolates (Uh4857-4 and Uh364) available in the NCBI (see text footnote 1) database were screened for SSR motifs (Supplementary Files S1, S2). The frequency of occurrence, relative density, and relative abundance of the repeats motifs was analyzed using Krait software (Du et al., 2018) with default settings. Thirty-five SSR primers (Supplementary Table S1) present in both genomes were randomly selected for PCR amplification. Primers were generated using online PRIMER3 software^2^.

### Collection and Isolation of *U. hordei* Isolates

**FIGURE 1:**
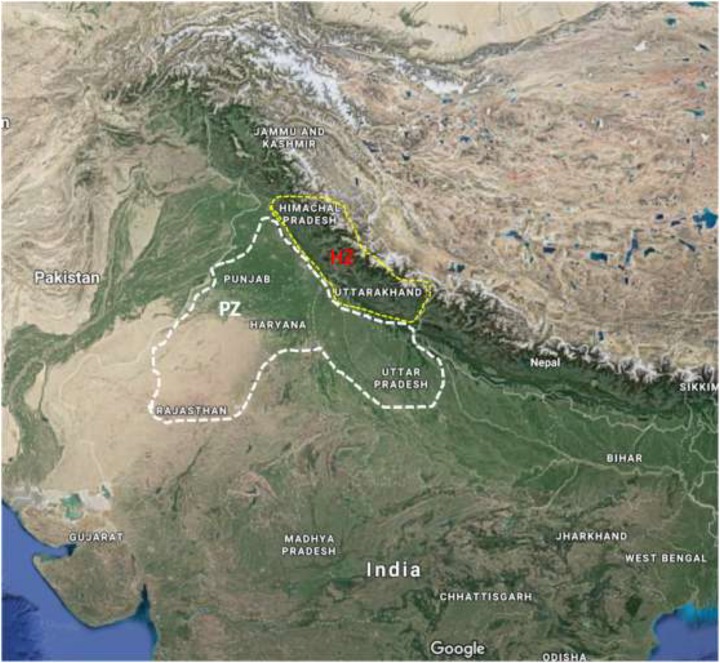
A map showing states of PZ (Uttar Pradesh, Punjab, Haryana, and Rajasthan) and HZ (Himachal Pradesh and Uttarakhand) surveyed for the collection of *Ustilago* hordei isolates from barley fields. PZ, plain zone; HZ, hill zone.

All *U. hordei* isolates were obtained from infected barley heads from two geographically and environmentally distinct areas (Figure 1 and Supplementary Table S2). One of these regions lies within the plain zone (PZ) of India composed of four states (Haryana, Punjab, Uttar Pradesh, and Rajasthan). Generally, barley in this zone is planted in late October–November and harvested in March end to mid-April. Moreover, barley varieties cultivated under this region is significantly different from the HZ region (Singh et al., 2014b). HZ covers the humid Himalayan regions (Siwalik Hills) situated beyond 750 m mean sea level and is composed of two states (Himachal Pradesh and Uttarakhand). This zone is characterized by cool climate and longer growing season. Moreover, barley cultivation is generally performed during the months of October and November, while harvested during the months of May and June in this zone.

The single teliospore isolation method of McCluskey and Mills (1990) with minor modifications was adopted to raise the culture of *U. hordei* from single smutted barley spike per field and designated as an isolate. Briefly, teliospores from individual smutted ear were scraped off from the spike and dried at 37°C for 4 h. From each smutted spike, 10 mg of teliospore material was weighed and suspended in 1000 μl of sterile saline solution (0.85 M NaCl) containing streptomycin sulfate (0.05 g L^–1^) and incubated for 30 min at 25 ± 2°C. Later, 100 μl of the teliospore spore suspension of each serial dilution (up to 10^–5^) was smeared on PDA medium for teliospore germination. Single germinating teliospore was transferred to slants containing 50% PDA (Hi Media, India), incubated at 25 ± 2°C for 3 days and stored at 4°C for further use.

### Total Genomic DNA Extraction

Each *U. hordei* isolate was grown in liquid potato-dextrose broth (50 ml) in a conical flask (250 ml) at 25 ± 2°C with shaking at 150 rpm. After 7 days, fungal mycelium was harvested and immediately ground into a fine powder in liquid nitrogen for DNA extraction according to the procedure adopted by Singh et al. (2014a). DNA concentrations were estimated using a Biodrop Spectrophotometer. DNA was routinely diluted to 1:10 (v:v) in Tris EDTA buffer before performing polymerase chain reaction (PCR).

### Microsatellite Genotyping

The amplification for each microsatellite marker was carried out in a gradient PCR (Q cycler 96, Hain Lifescience, United Kingdom) to determine the best annealing temperature (Ta). For each SSR primer set, PCR was performed in 10 μl of total volume containing GoTaq^®^ Green Master Mix (Promega), 0.5 pmol of each forward and reverse primer (Supplementary Table S1 and Table 1), and 25 ng of genomic DNA. PCR reaction without template DNA was employed as a no template control (NTC). DNA amplifications were carried out in Q cycler 96 (Hain Lifescience, United Kingdom) using the following thermal cycling parameters: initial denaturation (94°C for 4 min), followed by 35 cycles of denaturation (94°C for 60 s), annealing at temperatures corresponding to each primer pair as mentioned in Table 1 for 1 min, extension at 72°C for 1 min, and a final extension step at 72°C for 7 min. PCR products were electrophoretically discriminated on 3% agarose gel stained with ethidium bromide and visualized under ultraviolet (UV) light. Amplicon size was determined by making comparison with a 100-bp DNA ladder (Promega). For each SSR primer pair, amplicons of the same size across different isolates were considered to be the same allele.

**TABLE 1:** Genetic characteristics of 15 polymorphic simple-sequence repeat (SSR) loci in 59 Indian isolates of *U. hordei.*

**Locus**	**Primer sequence (5′–3′)**	**Repeat motif**	**Amplicon size (bp)**	**Amplicon range**	**T_a_ (°C)**	**Polymorphism (%)**	**N_a_**	**H_e_**	**PIC (%)**
UHB3	TAGCCTTTGAGGTCGATGTAGG	(GTT)_5_	246	225–250	54	100	2	0.0997	0.0948
	TGGGTGTCTTTCAGATGAGTTG								
UHB4	CAATTACTCGTCGTCCTCCTTC	(TCC)_6_	222	200–225	54	100	2	0.1349	0.1258
	AGCGTTCAGCGTAAGGTAGTTC								
UHB5	GCTCGTCTACCTCTGCGATACT	(GGA)_5_	241	240–280	55	100	2	0.1769	0.1612
	TCTGCATCTCAATCAACCAATC								
UHB6	AGACATGCACCGTAACAACAAC	(CGG)_6_	204	210–240	55	100	2	0.4767	0.3631
	TACCCTCCATACTCTTGTCCGT								
UHB17	TCTTGTGGAGTCTGCTGTTGTT	(TGC)_5_	239	220–250	54	100	2	0.2688	0.2327
	GTAGCTTCAGGTCGCATCACTT								
UHB 20	GGTTGTTGTCATAGGGGTTGTC	(TCG)_4_	259	260–520	54	100	2	0.4842	0.367
	TACCAGAACATGGGTTTCAGC								
UHB21	AGGTCTGGTGTGAGTGTTGATG	(TGA)_4_	258	225–260	54	100	2	0.233	0.2058
	CTCCTCATTGTAGTGCGTGTGT								
UHF 25	TACTTCTCCTCCTCCTCCTCCT GAACTCGCAAAGTGGTTTCTCT	(TATT)_5_	285	300–350	52	100	2	0.4904	0.3702
UHB 26	AGAGACCAAGTCGAATCCAAAG CCTTGCCTACTTCTCCCTACCT	(CAAGG)_6_	294	260–300	52	100	2	0.3866	0.3119
UHB 27	CATTTCAGTGTTGGACAAGCAT AGAGAGTTTCGTAGTTGGGCAG	(GTGTCA)_4_	251	200–250	52	100	2	0.2408	0.2118
UHB 28	CTAAGCATAAGGAGGCAACCAG	(TAAAA)_5_	271	280–850	54	100	2	0.4228	0.3334
	CGGAGTATTGGGAGTGAAATGT								
UHB 30	GGTGATTGGAAGACCACAGAAT GTTTTGAACTCTCTGCTTTGGG	(AAGCCA)_5_	227	180–230	55	100	2	0.2726	0.2354
UHB 31	CACAAACACACACACACACACA	(GCTCCC)_4_	224	200–230	54	100	2	0.375	0.3047
	CTGAACAGTAAAGCCTGAAGGG								
UHB 32	TCCTACATTGGGATGACTGATG GACTCGCTTCTTGTTCTTGGTT	(CAACGG)_4_	217	190–220	52	100	2	0.2008	0.1806
UHB 36	ATGAGGTCAAGAGTCAGCAACA ATTCGTCAAGATGCCTTTCACT	(GA)_8_	200	180–210	54	100	2	0.2149	0.1918

*N_*a*_, number of alleles; T_*a*_, annealing temperature; H_*e*_, expected heterozygosity; and PIC, polymorphism information content.*

### Bioinformatic Tools and Molecular Data Analysis

The gels were scored for the presence or absence of prominent and reproducible amplicons. Each amplicon was designated as a locus with two alternative alleles. The data of SSR amplification in different isolates were transformed into a binary data matrix as discrete variables (0 = absence and 1 = presence). The cluster analysis was performed with the NTSYS version 2.1 program (Rohlf, 2002) based on Unweighted Pair Group Method with Arithmetic Mean (UPGMA) algorithm to understand partitioning variation between populations. Bootstrap value was generated by FreeTree software (Pavlícek et al., 1999). GenAlEx 6.5 program (Peakall and Smouse, 2012) was used to determine polymorphic bands (%), the numbers of effective alleles (Ne), Nei’s gene diversity index (*H*), and Shannon’s information index (*I*). The polymorphic information content (PIC) value for each SSR marker was calculated using the formula mentioned by Kashyap et al. (2019). POPGENE version 1.31 software (Yeh et al., 1999) was used to calculate the cumulative allelic diversity (Ht), mean allelic diversity within populations (Hs), the proportion of the total allelic diversity (Gst), and the gene flow (Nm) among populations. The hierarchical analysis of molecular variance (AMOVA) was performed using a computer-based GenAlEx 6.5 program (Peakall and Smouse, 2012). Population structure analysis was performed with STRUCTURE 2.3.4 (Pritchard et al., 2000). The most favorable number of populations (*K*) was selected by testing *K* = 1 to *K* = 15 using five independent runs of 25,000 burn in period length at fixed iterations of 100,000. The optimum *K*-value was standardized by following the methodology of Evanno et al. (2005). The standardized index of association (SIA) was computed using LIAN (Linkage Analysis) version 3.7 software (Haubold and Hudson, 2000) to dissect the linkage disequilibrium (LD) under the null hypothesis that alleles observed at different loci are unlinked.

## Results

### Detection and Distribution of SSRs in *U. hordei* Genome

A total of 100,239 and 137,442 mono- to hexa-nucleotide repeat microsatellite sequences were identified from 20.13 and 26.94 Mb of assembled genomic sequences of Uh364 and Uh4857-4 isolates of *U. hordei*, respectively (Table 2), with an average relative density of 82,253.64 and 85,178.03 bp per Mb and relative abundance of 5125.14 and 5246.27 microsatellites per Mb, respectively. A total of 72,865 and 98,613 SSRs with perfect motifs were detected in the Uh364 and Uh4857-4 genomes, respectively (Supplementary Figure S1). The maximum percent of SSRs among two genome sequence sets was of penta-nucleotide motifs (31.29 and 29.75%) followed by tri-nucleotide (28.27 and 29.88%), hexa-nucleotide (19.86 and 20.78%), mono-nucleotide (17.21 and 16.56%), di-nucleotide (1.8 and 1.59%), and tetra-nucleotides (1.56 and 1.45%) in both Uh364 and Uh4857-4 genomes, respectively (Supplementary Figure S2 and Supplementary Table S3). Microsatellites were categorized into class I consisting of perfect core motifs above 20 bp and class II consisting of 10- to 20-bp-long motifs (Supplementary Figure S3). The number of perfect microsatellites assigned to class I in both the genomes Uh364 and Uh4857-4 was 2049 and 2684, respectively. Similarly within class II, 43,090 and 58,947 SSRs were found in both Uh364 and Uh4857-4 genomes, respectively (Supplementary Figure S3). The most frequent motif in Uh364 and Uh4857-4 genome was CT_86_ and CAA_73_ followed by TGT_43_ and T_71_, respectively (Supplementary Table S3). Overall, the repeats of A, T, CT, TG, TGT, CAA, AAGC, ATTT, ATAA, TGCT, CTTTT, AAATC, CCCTCG, and CCCTAA were remarkably abundant in both genomes. The A/T motif in both genomes (Uh364 and Uh4857-4) was the most abundant in mono-nucleotide motif (12.19 and 12.08%, respectively). With respect to the di-nucleotide motif, AG/CT was the most abundant type, with a total of 664 (0.91%) and 838 (0.85%) in both genomes, respectively. A total of 5843 and 6691 tri-nucleotide motif types in both genomes were identified with ACG/CGT, representing approximately 8.02 and 6.79%, respectively, while AAG/CTT accounted for 3.85 and 3.27%, respectively, in both genomes (Table 3). The hexa-nucleotide motif, AAAAAG/CTTTTT, covered 0.53 and 0.52% portion of Uh364 and Uh4857-4 genome, respectively (Table 3).

**TABLE 2:** Number and distribution of SSRs in whole genome of two isolates of *U. hordei.*

**Parameter/isolate**	**Uh364**	**Uh4857-4**
NCBI BioProject No.	PRJNA395628	PRJEA79049
NCBI GenBank assembly accession	GCA_003012045.1	GCA_000286035.1
Whole Genome Release date	20.03.2018	20.12.2013
Total size covered by examined sequences (Mb)	20.13	26.94
Number of SSR identified	100,239	137,442
Perfect SSRs	72,865	98,613
Imperfect SSRs	21,633	31,665
Compound SSRs	5741	7164
Total length of SSRs	1,655,993	2,294,817
Relative density (bp per Mb)	82,253.64	85,178.03
Relative abundance (SSR per Mb)	5125.14	5246.27

**TABLE 3:** Comparative account of total count length, percentage, relative abundance, and relative density of most abundant motifs in the *U. hordei* genome.

**Motif**	**Counts**		**Length**		**Percent**		**Average length**		**Relative abundance**		**Relative density**	
	**Uh364**	**Uh4857-4**	**Uh364**	**Uh4857-4**	**Uh364**	**Uh4857-4**	**Uh364**	**Uh4857-4**	**Uh364**	**Uh4857-4**	**Uh364**	**Uh4857-4**
A/T	8879	11,910	71,432	100,935	12.19	12.08	8.05	8.47	441.02	442.07	3548.05	3746.46
ACG/CGT	5843	6691	58,590	66,888	8.02	6.79	10.03	10	290.22	248.35	2910.18	2482.72
C/G	3662	4422	25,275	30,018	5.03	4.48	6.9	6.79	181.89	164.13	1255.42	1114.2
AAG/CTT	2805	3224	27,429	31,446	3.85	3.27	9.78	9.75	139.33	119.67	1362.41	1167.2
ACC/GGT	2450	2815	24777	28,347	3.36	2.85	10.11	10.07	121.69	104.49	1230.68	1052.17
AAC/GTT	1522	2053	17,598	22,617	2.09	2.08	11.56	11.02	75.6	76.2	874.1	839.49
AAACG/CGTTT	814	994	8390	10,275	1.12	1.01	10.31	10.34	40.43	36.89	416.73	381.38
AAAAG/CTTTT	775	895	8680	10,105	1.06	0.91	11.2	11.29	38.49	33.22	431.14	375.07
AG/CT	664	838	12,502	15,328	0.91	0.85	18.83	18.29	32.98	31.1	620.98	568.94
AC/GT	501	540	10,218	10,816	0.69	0.55	20.4	20.03	24.88	20.04	507.53	401.46
AAAAAG/CTTTTT	384	514	6102	8778	0.53	0.52	15.89	17.08	19.07	19.08	303.09	325.82
AAT/ATT	291	419	3465	4896	0.4	0.42	11.91	11.68	14.45	15.55	172.11	181.73
AAAAC/GTTTT	285	349	3060	3725	0.39	0.35	10.74	10.67	14.16	12.95	151.99	138.26
AAACC/GGTTT	243	300	2445	3015	0.33	0.3	10.06	10.05	12.07	11.14	121.44	111.91
AAAAT/ATTTT	199	258	2275	2910	0.27	0.26	11.43	11.28	9.88	9.58	113	108.01
AACG/CGTT	174	196	2736	2996	0.24	0.2	15.72	15.29	8.64	7.28	135.9	111.2
AT	138	176	2464	3022	0.19	0.18	17.86	17.17	6.85	6.53	122.39	112.17
AAAG/CTTT	100	131	1452	1864	0.14	0.13	14.52	14.23	4.97	4.86	72.12	69.19
AAAACG/CGTTTT	91	115	1152	1596	0.12	0.12	12.66	13.88	4.52	4.27	57.22	59.24
AAAAAT/ATTTTT	88	110	1230	1464	0.12	0.11	13.98	13.31	4.37	4.08	61.09	54.34
AAAAAC/GTTTTT	78	107	1362	1680	0.11	0.11	17.46	15.7	3.87	3.97	67.65	62.36
AAAT/ATTT	56	79	960	1252	0.08	0.08	17.14	15.85	2.78	2.93	47.68	46.47
AACC/GGTT	42	49	576	660	0.06	0.05	13.71	13.47	2.09	1.82	28.61	24.5
AAAC/GTTT	35	37	448	484	0.05	0.04	12.8	13.08	1.74	1.37	22.25	17.96
AAAACC/GGTTTT	33	36	444	498	0.05	0.04	13.45	13.83	1.64	1.34	22.05	18.48
CG	9	10	112	124	0.01	0.01	12.44	12.4	0.45	0.37	5.56	4.6

### Polymorphism of SSR Markers

To determine the allelic diversity among *U. hordei* isolates, 50 randomly selected SSR primers present in both genomes were employed. Thirty-five SSR primer pairs displayed prominent amplification of at least single amplicon while the remaining primers did not show any amplification. Out of 35, only 15 primers (Table 1) revealed polymorphism and therefore employed further for analyzing genetic studies of 59 isolates of *U. hordei* collected from barley fields of hilly (HZ) and plain (PZ) terrains. The polymorphism data for each locus are mentioned in Table 1. An average of two alleles per microsatellite was generated with band size ranging from 180 to 850 bp. PIC was varied between 0.095 and 0.37 (Table 1). Ewens–Watterson test indicated that all the tested markers are of neutral type (Supplementary Table S4).

### Population Genetic Diversity Analysis

Genetic analysis revealed that effective alleles (N_e_) and expected heterozygosity (H_e_) across all 15 loci in the whole population were varied from 1.3221 to 2.8792 and 0.0997 to 0.4904, respectively (Supplementary Table S5). The quantitative estimates of various genetic diversity indices for two distinct populations of *U. hordei* from barley-growing regions are shown in Table 4. Higher *N*_a_-value (1.433) was observed in the PZ population, while a lower value (1.066) was recorded in the HZ population (Table 4). In terms of expected heterozygosity (H_e_) and effective alleles (N_e_), the HZ population showed the least values (H_e_ = 0.1384; N_e_ = 0.766) relative to the PZ population (H_e_ = 0.2696; N_e_ = 1.140). Similarly, unbiased gene diversity (_u_H_e_) was the least in the PZ population (_u_H_e_ = 0.5271) compared to the HZ population (_u_H_e_ = 0.5903). The polymorphic loci (%) were varied from 86.67 (HZ) to 100% (PZ), with an average of 93.34% (Table 4). No private allele was detected in the entire population.

**TABLE 4:** Summary of the population diversity indices and estimation of linkage disequilibrium computed on the basis of SSR markers.

**Population**	***N***	**N_a_**	**N_e_**	**I**	**H_e_**	**_u_H_e_**	**PL (%)**	**SIA (*P*-value)**	***V*_D_ > *L***	**LD or LE**
HZ	38	1.066 ± 0.319^∗^	0.766 ± 0.279	0.227 ± 0.167	0.1384 ± 0.1083	0.5903	86.67	0.022 (<0.001)	Yes	LD
PZ	21	1.433 ± 0.175	1.140 ± 0.259	0.4425 ± 0.0920	0.2696 ± 0.0536	0.5271	100	0.129 (<0.001)	Yes	LD
Total	59							0.181	Yes	LD

**N*, number of isolates; N_*a*_, number of different alleles; N_*e*_, number of effective alleles = 1/(p2 + q2); *I*, Shannon’s information index; H_*e*_, expected heterozygosity = 2 × *p* × *q*; PL (%), percent polymorphic loci. ^∗^The values after ± denote the standard errors. PZ, plain zone; HZ, Hill zone. Standardized index of association (SIA), *V*_*D*_, and *V*_*e*_ were calculated using LIAN version 3.5 software. *P*-values were estimated from 1000 randomizations. *V*_*D*_, variance of pairwise differences; *L*, 95% critical value for VD; LE, linkage equilibrium; LD, linkage disequilibrium.*

### Genetic Differentiation

The results of AMOVA (Supplementary Table S6) revealed distribution of substantial genetic variation within *U. hordei* isolates (87%); however, genetic variation among populations was low (13%). Pairwise comparison between HZ and PZ populations reflected very low but significant genetic distance values (*P* < 0.001). The average and constant level of gene flow (Nm = 1.009) was noticed between HZ and PZ populations. Pairwise comparison of HZ and PZ showed a genetic identity of 0.198 levels (Supplementary Table S7).

### Population Genetic Structure

**FIGURE 2:**
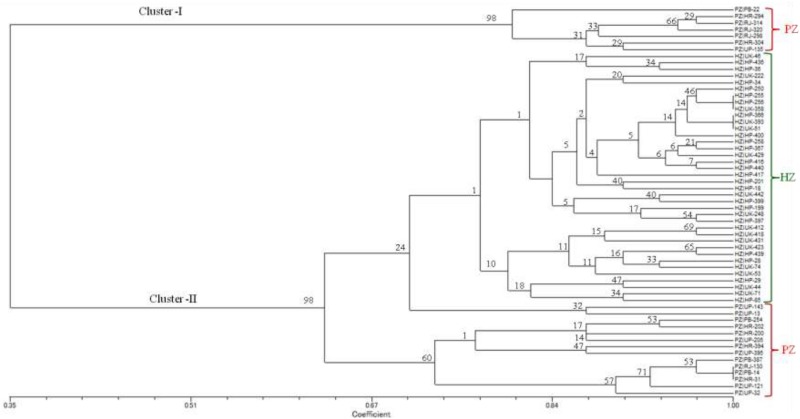
UPGMA dendrogram from SSR data for 59 isolates of *Ustilago hordei* amplified by 15 microsatellites. Scale indicates Jaccard’s coefficient of similarity. The numbers at the branches are confidence values based on Felsenstein’s bootstrap produced by FreeTree software (Pavlícek et al., 1999). HZ, hill zone; PZ, plain zone.

The dendrogram based on UPGMA displayed spatial clustering and divided all the 59 isolates of *U. hordei* into two distinct groups at the similarity coefficient level of 0.65 (Figure 2). Cluster I consisted of seven isolates (PB-22, HR-294, RJ-314, RJ-320, RJ-298, HR-304, and UP-135), which were collected from PZ representing Punjab, Haryana, Rajasthan, and Uttar Pradesh states. Several sub-groups were noticed in cluster II, indicating genetic variability within and among isolates of both zones. Cluster II includes 38 isolates representing HZ and were collected from Uttarakhand (UK-46, UK-222, UK-358, UK-412, UK-418, UK-423, UK-429, UK-431, UK-442, UK-248, UK-393, UK-44, UK-51, UK-53, UK-71, and UK-74) and Himachal Pradesh (HP-250, HP-255, HP-256, HP-258, HP-416, HP-417, HP-436, HP-439, HP-440, HP-36, HP-199, HP-201, HP-366, HP-367, HP-397, HP-399, HP-400, HP-28, HP-29, HP-34 HP-85, and HP-18) states.

There was a sub-cluster of 14 isolates (UP-143, UP-13, PB-254, HR-202, HR-200, UP-205, HR-394, UP-395, PB-387, RJ-130, PB-14, HR-31, UP-121, and UP-32) grouped into cluster II, representing Punjab, Haryana, Rajasthan, and Uttar Pradesh states.

**FIGURE 3:**
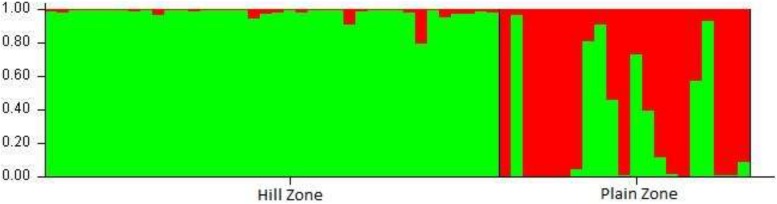
Population structure analysis of 59 isolates of *Ustilago hordei*. Analysis was carried out using STRUCTURE software with *K* set at 2. Inferred ancestries of the 59 UH isolates were based on two geographical distinct population.

Structure analysis performed to determine the genetic relationship among *U. hordei* isolates, excluding loci with null alleles, demonstrated a strong signal with a single clear peak at *K* = 2 (Figure 3). Moreover, *K* = 2 indicated the existence of two genetically distinct groups within the 59 studied *U. hordei* isolates.

### Linkage Disequilibrium

The LD analysis reflected positive but low SIA values (0.0229–0.129) that deviated from zero in the case of both HZ and PZ populations (Table 4). When isolates of *U. hordei* representing HZ and PZ were evaluated as a single population, a SIA value of 0.181 (*P* < 0.001) and a *V*_D_-value higher than the *L*-value was observed, advocating a state of LD (Table 4).

## Discussion

The whole genome data of two isolates of *U. hordei* (Uh4857-4 and Uh364) have been released in 2013 and 2018, respectively, but, still these have not been elucidated for distribution, abundance, and frequency of microsatellite motifs. To the best of our knowledge, the present investigation provides the first comprehensive information about the microsatellite distribution and their comparison between assembled genomes of both the *U. hordei* isolates. Further, 15 new microsatellite markers were developed for analyzing the genetic differentiation and population structure of 59 isolates of *U. hordei* collected from barley field representing two different terrains (HZ and PZ) in India. In earlier reports, a small number of microsatellites developed from *U. hordei* genome have been utilized for analyzing the genetic variability between the isolates of *U. hordei* in China (Yu et al., 2016). However, the current study was performed on the complete genome assembly of Uh4857-4 and Uh364 isolates and non-coding sequences were also included. As a consequence, it permits to report a huge number of microsatellites (100239 in Uh364 and 137442 in Uh4857-4) compared to earlier reports in phytopathogenic fungi (Kumar et al., 2012; Singh et al., 2014a; Zhang et al., 2015; Mahfooz et al., 2017; Choudhary et al., 2018; Varady et al., 2018). In the present study, the relative abundance of microsatellites in the whole genome of Uh4857-4 (5246.27 microsatellites per Mb) and Uh364 (5125.14 microsatellites per Mb) is quite similar, suggesting the highly homogeneous genomic structure of these two *U. hordei* isolates. Moreover, the pattern of microsatellite distribution in terms of motif type and repeat number is almost identical in assembled genome of both the isolates. The A/T-rich motifs are present in large numbers, while C/G-rich motifs are least dominated in both genomes, which is in agreement with earlier reports of Singh et al. (2011). This observed microsatellite expansion and variation might be due to replication slippage that happened during DNA replication and observed more frequently in A/T-rich motifs in comparison to C/G-rich motifs as reported earlier (Klintschar et al., 2004; Forster et al., 2015).

In the current investigation, two terrains, i.e., HZ and PZ, were chosen because they are geographically distinct from one another, differ greatly in size, and are subject to different barley cultivation and management practices. The results of the present study revealed low but significant genetic differentiation in *U. hordei* isolates with a similarity coefficient of over 60%. Parallel to these findings, Xu et al. (2004) also documented the phenomenon of diverse geographical origin of *U. scitaminea* population with small values of dissimilarity coefficients (Owati et al., 2019). The low level of genetic differentiation and formation of two distinct sub-groups within *U. hordei* isolates of PZ reflected an extreme event of genetic discrimination that happened at a small scale, probably due to migration. Moreover, it seems that probably in some of the areas of HZ, *U. hordei* isolates migrated from PZ through anthropogenic activities linked with barley seed production and distribution. This statement was firmly supported by the restricted and sympatric distribution of two distinct clusters (PZ and HZ) in the barley sampling fields and by the observation that two different barley-growing terrains were clustered in the same lineages. Therefore, the PZ population was most likely migrated into the HZ in recent times.

An AMOVA based of microsatellite markers revealed that 13% of the total molecular variance belonged to variation among *U. hordei* populations (*P* < 0.001), whereas the rest (87%) was due to variations within population. Similarly, low variation (16%) among population of *U. maydis* and comparatively more variation (84%) within *U. maydis* population were observed in Mexico (Jiménez-Becerril et al., 2018). On parallel lines, Owati et al. (2019) reported 85% of the total diversity within the population of *Didymella pisi*, and 15% of the total genetic diversity among populations in Montana. Thus, a major portion of total molecular variance that existed within the population, as observed in the present study, could be due to the relatively moderate level of gene flow (Nm = 1.009) between the *U. hordei* population (geographic distance between populations of 150–800 km). Therefore, migration plays a more important role than genetic drift in the case of the present study. It is also a well-established fact that in the sampling sites, the teliospores of CS fungus are proficient for travel by wind and human interventions, resulting in minimizing the genetic differences among populations. Moreover, there is also the possibility that the genetic differences in the *U. hordei* population may be influenced by environmental conditions, geography, and cultivation of different types of barley cultivars. Overall, it seems that gene flow mediated processes as a consequence of anthropogenic activities, such as movement and exchange of infected plant material responsible for causing significant variation among a pathogen population.

There are several reports that describe low level of genetic variation in basidiomycetes fungi (Hamelin et al., 1995; Braithwaite et al., 2004; Zhou et al., 2008). Similarly, the average similarity coefficient was 0.65, demonstrating a moderate level of genetic variation among all the studied isolates. This may be attributed to the regular exchange of barley cultivars of PZ to HZ areas in recent time owing to the fact that *U. hordei* is a seed- and soil-borne fungus. LD analysis conducted to explore the extent of recombination within *U. hordei* population indicated positively significant but relatively low SIA value deviating extensively from zero in both the population sets. This clearly indicated a state of LD in both PZ and HZ populations, which might be due to the presence of epidemic isolates (i.e., isolates exhibiting strong competitive potential). These results are in line with the earlier reports of Lomholt et al. (2001); Haubold and Hudson (2000), Morrison et al. (2008), and Kashyap et al. (2019). The current study provides the baseline information, and a large sample size coupled with ecology data of a wider geographical area in HZ and PZ is still required to validate the LD state of the *U. hordei* population. Moreover, Bayesian clustering and UPGMA analysis based on polymorphic SSR markers also divided *U. hordei* population into two distinct genetic clusters. However, 23.72% isolates were found as admixture. These observations were similar to the earlier findings of Yu et al. (2016), where they reported molecular variation and differentiation associated with geographical origin excluding few isolates of *U. hordei* belonging to Tibetan areas of China. Structure analysis in the present study revealed two clusters (*K* = 2 with microsatellite data) and pointed toward the existence of significant structure in *U. hordei* populations. The population structure is composed of sub-groups formed as a result of admixture of isolates representing PZ isolates in HZ cluster and thereby highlighting a common origin source for *U. hordei* populations or the role of migration and gene flow among *U. hordei* populations.

The microsatellite markers generated in the current investigation are very useful to decipher the genetic variability and population structure of *U. hordei* isolates from barley fields of India. Despite geographical barriers (HZ and PZ), the observed genetic variations in the *U. hordei* population suggest frequent exchange of planting materials and dispersal of inocula among the barley growing regions. Migration of *U. hordei* isolates between barley-growing regions appeared accidental through movement of infected materials or though barley breeding linked processes. Overall, this study provides baseline information that can be further utilized to dissect evolutionary history and pathogen biology for in-depth analysis of disease dynamics, plant–fungus interactions, and development and deployment of resistant varieties and evolve effective disease management module. Moreover, genomic resources generated from this study can be used to develop novel specific primers for identification and molecular characterization of *U. hordei.*

## Data Availability Statement

Publicly available datasets were analyzed in this study. This data can be found here: https://www.ncbi.nlm.nih.gov/genome/?term=ustilago+hordei.

## Author Contributions

PK and SK conceived and designed the work, and drafted the manuscript. PK, SK, and PJ performed the sampling survey. RK, RT, PK, AS, and PS conducted the experiments. RT, PJ, and AS performed the data analysis. SK and GS performed the final editing and proofing of the manuscript. All authors approved the submitted version of the manuscript.

## Conflict of Interest

The authors declare that the research was conducted in the absence of any commercial or financial relationships that could be construed as a potential conflict of interest.

## Footnotes

1 www.ncbi.nlm.nih.gov

2 http://bioinfo.ut.ee/primer3-0.4.0/
