# Supplementary material for: Identification of Novel Microsatellite Markers to Assess the Population Structure and Genetic Differentiation of Ustilago hordei Causing Covered Smut of Barley
Source: Front Microbiol. 2020 Jan 15;10:2929. doi: 10.3389/fmicb.2019.02929 (PMC6974468; doi:10.3389/fmicb.2019.02929)
Supplement: Supplementary file 1 [file Data_Sheet_2.ZIP › Supplementary file/Supplementary figures.docx]

**
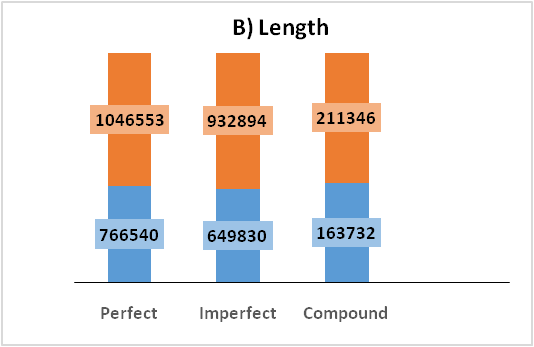

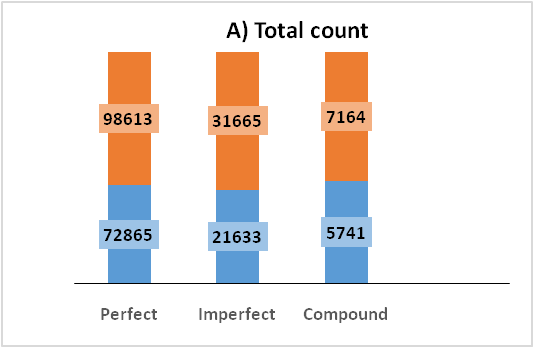
**

**
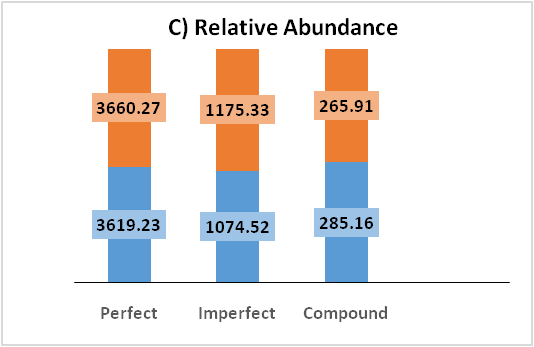

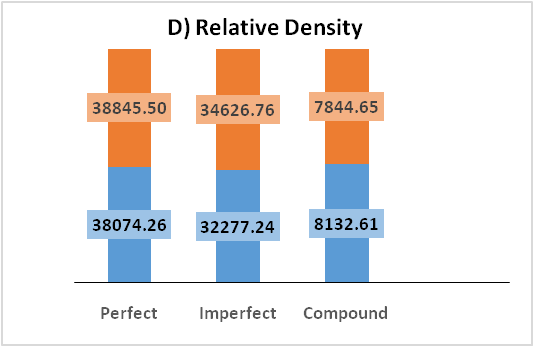
**

**Fig S1:** Comparative graphical account of total count (A), length (B), relative abundance (c), and relative density (D) of SSRs in whole genome sequence of Uh364 (blue bar) and Uh4857-4 (orange bar) isolates.


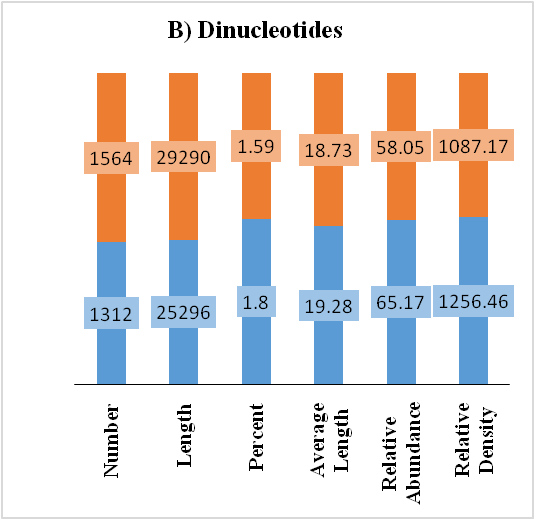

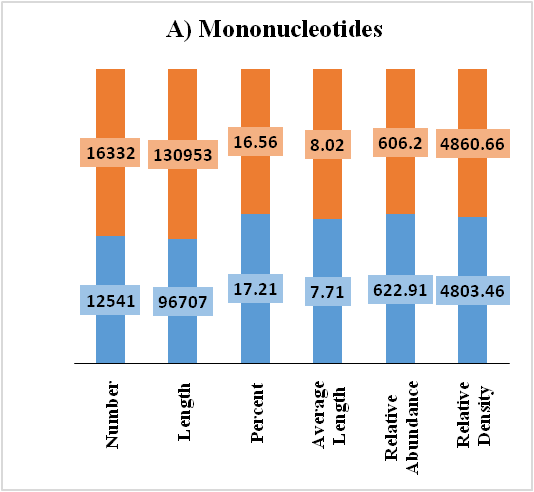


**
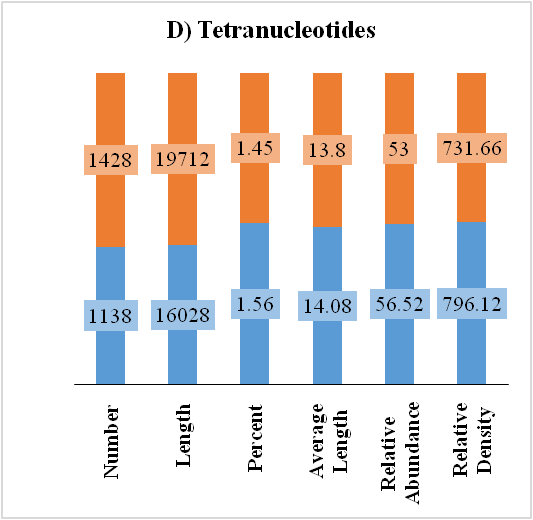

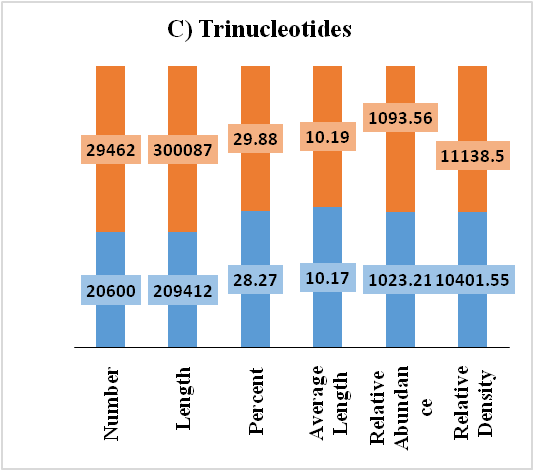
**

**
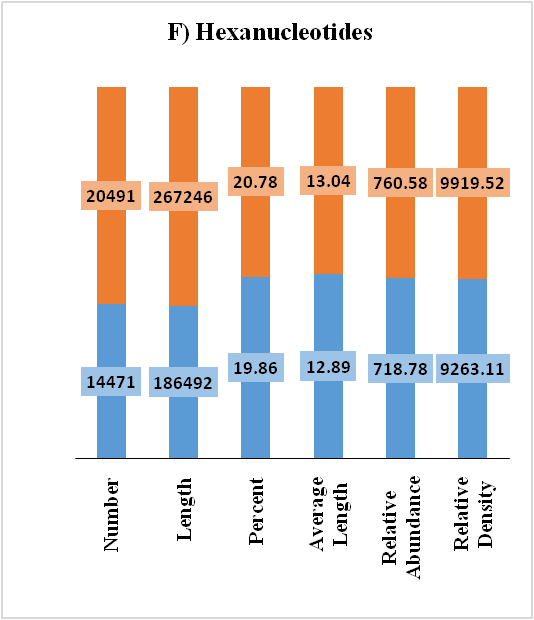

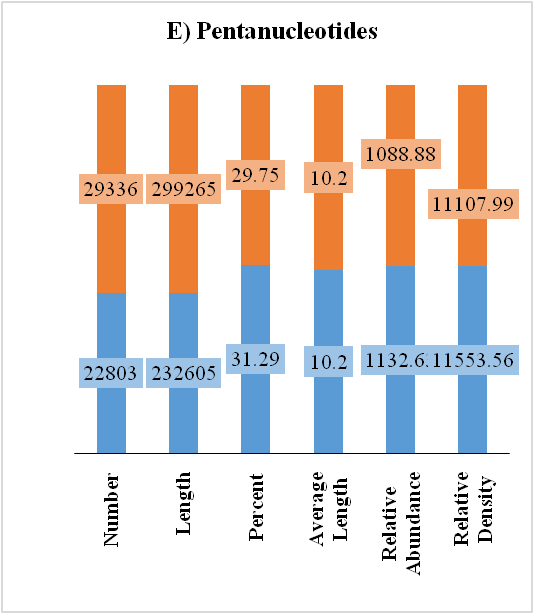
**

**Fig S2:** Graphical representation of sharing of different classes of motifs in the whole genome sequence of Uh364 (blue bar) and Uh4857-4 (orange bar) isolate


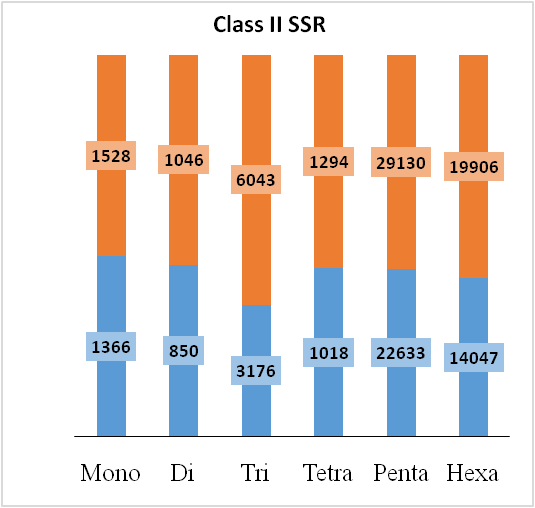

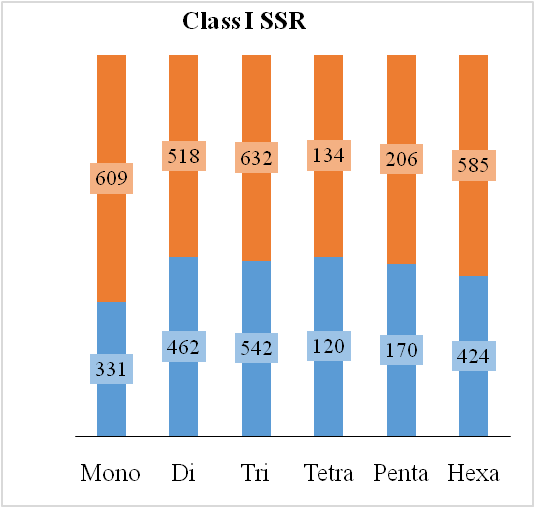


**Fig S3:** Distribution of Class I (A) and class II (B) repeat motifs in the whole genome sequence of Uh364 (blue bar) and Uh4857-4 (orange bar) isolate. Class I SSR = Length ≥ 20; Class II SSR= Length 10 ≤ Length < 20
