# Supplementary material for: Identification of Novel Microsatellite Markers to Assess the Population Structure and Genetic Differentiation of Ustilago hordei Causing Covered Smut of Barley
Source: Front Microbiol. 2020 Jan 15;10:2929. doi: 10.3389/fmicb.2019.02929 (PMC6974468; doi:10.3389/fmicb.2019.02929)
Supplement: Supplementary file 1 [file Data_Sheet_2.ZIP › Supplementary file/Supplementary Tables.docx]

| **Table S1:**  Thirty five SSR primers selected as random microsatellites markers present in both genomes for preliminary screening of polymosphism of *U. hordei* isolates | | | | | | |
| --- | --- | --- | --- | --- | --- | --- |
| **S. No.** | **Motif** | **Forward Primer** | **Tm** | **Reverse Primer** | **T_m_** | **Predicted amplicon size** |
| UHB1 | (TGC)6 | CCTCCTCATCGTACTCCTCATC | 60.10 | CTAGAATGGATCTCTCGCCCT | 59.82 | 270 |
| UHB 2 | (TCC)5 | GCAACAACAGAAGGACAGTGG | 60.74 | GGTTGATCGAAAAGATGTCTCC | 59.95 | 268 |
| UHB 3 | (GTT)5 | TAGCCTTTGAGGTCGATGTAGG | 60.62 | TGGGTGTCTTTCAGATGAGTTG | 60.15 | 246 |
| UHB 4 | (TCC)6 | CAATTACTCGTCGTCCTCCTTC | 60.13 | AGCGTTCAGCGTAAGGTAGTTC | 59.98 | 222 |
| UHB 5 | (GGA)5 | GCTCGTCTACCTCTGCGATACT | 60.07 | TCTGCATCTCAATCAACCAATC | 60.08 | 241 |
| UHB 6 | (CGG)6 | AGACATGCACCGTAACAACAAC | 59.97 | TACCCTCCATACTCTTGTCCGT | 59.89 | 204 |
| UHB 7 | (CAT)5 | AAGCATACTCAAGGCAGGGTAA | 60.15 | GTTCTCGGATGGTCTCGTCTAC | 60.14 | 235 |
| UHB 8 | (AGG)6 | AGACCATCGTACCGAGAAATTG | 60.37 | ACCTGACACCGAACTCTTGACT | 60.21 | 239 |
| UHB 10 | (TGA)5 | ACCTATTCAAGCAAGTGGTCGT | 60.06 | AGAGCAAACCAAGAGTCCAAAG | 59.92 | 254 |
| UHB 11 | (CAG)5 | ACGAGCAATCTTTCACCAGAAC | 60.67 | AGCCAAAGTCTGAATAGCCTGA | 60.39 | 219 |
| UHB 12 | (TGC)5 | GAAGAGGAAGGCTCAGACGATA | 59.98 | ATGGAAGAACAGAGACAGGGAA | 60.11 | 218 |
| UHB 13 | (GAG)6 | AAGGAGGAGAACTTGACCATGA | 60.11 | GTCTGTGTGTGTGCTGGTGTC | 60.28 | 234 |
| UHB 14 | (CCG)6 | AAAAGTCATCCTCGTTTCGGTA | 60.00 | AGATAGGGAAGCAAATCATGGA | 59.93 | 235 |
| UHB 16 | (GAC)6 | GCCTCTTCATCTCTCTCCTCAC | 59.59 | TGACTCTTCTGCATCATATCGG | 60.24 | 261 |
| UHB 17 | (TGC)5 | TCTTGTGGAGTCTGCTGTTGTT | 59.95 | GTAGCTTCAGGTCGCATCACTT | 60.82 | 239 |
| UHB 18 | (AGA)4 | GCTTTCTTTTCAGAGCGTTGAT | 60.03 | TCACTCTTCTCCTCTCCTCCAC | 59.99 | 219 |
| UHB 19 | (CTC)4 | GATACCTGTGCTCCGTCCATA | 59.97 | TCGACTATTTTGTTGGCAGTTG | 60.17 | 223 |
| UHB 20 | (TCG)4 | GGTTGTTGTCATAGGGGTTGTC | 60.52 | TACCAGAACATGGGTTTCAGC | 59.99 | 259 |
| UHB 21 | (TGA)4 | AGGTCTGGTGTGAGTGTTGATG | 60.07 | CTCCTCATTGTAGTGCGTGTGT | 60.24 | 258 |
| UHB 22 | (CAAG)4 | GTGTGCATCAACTTCCCATAAA | 59.87 | TTTCTCCCTTTTCATGTCCTGT | 59.98 | 274 |
| UHB 23 | (AAAT)4 | TCGTGAAAACTAACAGAGCCAA | 59.92 | ACACCTATTTGCGTGAAGGAGT | 60.06 | 220 |
| UHB 24 | (AACC)4 | TTCTCACATTCTCCTTCTGCAA | 59.99 | AAGTGGTTAGCTGAGTTGAGGG | 59.81 | 276 |
| UHB 25 | (TATT)5 | TACTTCTCCTCCTCCTCCTCCT | 59.85 | GAACTCGCAAAGTGGTTTCTCT | 59.93 | 285 |
| UHB 26 | (CAAGG)6 | AGAGACCAAGTCGAATCCAAAG | 59.75 | CCTTGCCTACTTCTCCCTACCT | 60.14 | 294 |
| UHB 27 | (GTGTCA)4 | CATTTCAGTGTTGGACAAGCAT | 60.04 | AGAGAGTTTCGTAGTTGGGCAG | 59.94 | 251 |
| UHB 28 | (TAAAA)5 | CTAAGCATAAGGAGGCAACCAG | 60.27 | CGGAGTATTGGGAGTGAAATGT | 60.24 | 271 |
| UHB 29 | (GATTT)4 | CTTGTGCTTGTTGTAGGTCGAG | 59.97 | TTTCCCTATTTTCCCGCTG | 60.02 | 285 |
| UHB 30 | (AAGCCA)5 | GGTGATTGGAAGACCACAGAAT | 60.23 | GTTTTGAACTCTCTGCTTTGGG | 60.28 | 227 |
| UHB 31 | (GCTCCC)4 | CACAAACACACACACACACACA | 60.05 | CTGAACAGTAAAGCCTGAAGGG | 60.29 | 224 |
| UHB 32 | (CAACGG)4 | TCCTACATTGGGATGACTGATG | 59.81 | GACTCGCTTCTTGTTCTTGGTT | 59.93 | 217 |
| UHB 33 | (GGAGAA)4 | GAAAGAGAGAGGGAGGGAAGAG | 59.96 | TGCGTATAGGTATGTGTGGCTT | 59.56 | 230 |
| UHB 34 | (CA)8 | GAAGAAAATGCTAGAGCGAAGG | 59.65 | AGCAGAAGGTGAGAGAGCGTAT | 59.69 | 216 |
| UHB 35 | (AG)9 | ATGAAGAGTACCAAGCCAAGGA | 60.13 | AAGCTCAACTCACAGCCAAAAT | 60.30 | 275 |
| UHB 36 | (GA)8 | ATGAGGTCAAGAGTCAGCAACA | 59.91 | ATTCGTCAAGATGCCTTTCACT | 60.14 | 200 |

| **Table S2:**  Geographical origin, source and year of collection of *U. hordei* isolates from barley fields | | | | | | |
| --- | --- | --- | --- | --- | --- | --- |
| **S. No.** | **Isolate code** | **Collection site(s)** | **Latitude** | **Longitude** | **Terrain type** | **Year of collection** |
| 1 | PB-22 | Bibi Wala, Punjab | 30.253784 | 74.993960 | PZ | 2015-16 |
| 2 | UK-46 | Jagjeetpur, Uttrakhand | 29.915014 | 78.118891 | HZ | 2015-16 |
| 3 | UK-222 | Lusipur, Uttrakhand | 30.328598 | 77.932821 | HZ | 2015-16 |
| 4 | HP-250 | Balh, Himachal Pradesh | 31.952529 | 77.077988 | HZ | 2015-16 |
| 5 | PB-254 | Kutlupur, Punjab | 31.317788 | 75.521428 | PZ | 2015-16 |
| 6 | HP-255 | Gramang, Himachal Pradesh | 31.954838 | 77.028647 | HZ | 2015-16 |
| 7 | HP-256 | Tikra Babli, Himachal Pradesh | 31.940482 | 77.112741 | HZ | 2015-16 |
| 8 | HP-258 | Sultanpur, Himachal Pradesh | 31.965998 | 77.102974 | HZ | 2015-16 |
| 9 | HR-294 | Sasauli, Haryana | 30.150306 | 77.258640 | PZ | 2015-16 |
| 10 | HR-304 | Azad Nagar, Haryana | 29.129698 | 75.693159 | PZ | 2015-16 |
| 11 | RJ-314 | Durgapura, Rajasthan | 26.851713 | 75.793250 | PZ | 2015-16 |
| 12 | RJ-320 | Bikaner, Rajasthan | 27.988188 | 73.346219 | PZ | 2015-16 |
| 13 | UK-358 | Chalang, Uttrakhand | 30.388833 | 78.105321 | HZ | 2015-16 |
| 14 | PB-387 | Khawaspur, Punjab | 31.500354 | 75.907411 | PZ | 2015-16 |
| 15 | UK-412 | UAS Nagar, Uttrakhand | 29.019007 | 79.429661 | HZ | 2015-16 |
| 16 | HP-416 | Bahot, Himachal Pradesh | 31.522192 | 76.889486 | HZ | 2015-16 |
| 17 | HP-417 | Sundar Nagar, Himachal Pradesh | 31.536197 | 76.894743 | HZ | 2015-16 |
| 18 | UK-418 | Nagla, Uttrakhand | 28.996153 | 79.508140 | HZ | 2015-16 |
| 19 | UK-423 | Pattharchatta, Uttrakhand | 29.027509 | 79.412870 | HZ | 2015-16 |
| 20 | UK-429 | Phool Bagh, Uttrakhand | 28.993742 | 79.449070 | HZ | 2015-16 |
| 21 | UK-431 | Village Sarson, Uttrakhand | 29.592685 | 79.661713 | HZ | 2015-16 |
| 22 | HP-436 | Galore, Himachal Pradesh | 31.682296 | 76.518142 | HZ | 2015-16 |
| 23 | HP-439 | Himachal Pradesh | 31.680520 | 76.523569 | HZ | 2015-16 |
| 24 | HP-440 | Kohta, Himachal Pradesh | 31.691466 | 76.525304 | HZ | 2015-16 |
| 25 | UK-442 | Hawal Bagh, Himachal Pradesh | 29.614524 | 79.672457 | HZ | 2015-16 |
| 26 | HP-36 | Una, Himachal Pradesh | 31.495650 | 76.217494 | HZ | 2016-17 |
| 27 | HP-199 | Kangra, Himachal Pradesh | 31.898204 | 76.173185 | HZ | 2016-17 |
| 28 | HR-200 | Karnal, Haryana | 29.664413 | 77.023362 | PZ | 2016-17 |
| 29 | HP-201 | Kangra, Himachal Pradesh | 31.887846 | 76.198143 | HZ | 2016-17 |
| 30 | HR-202 | Karnal, Haryana | 29.708759 | 76.968313 | PZ | 2016-17 |
| 31 | UP-205 | Mau, Uttar Pradesh | 25.933049 | 83.555467 | PZ | 2016-17 |
| 32 | UK-248 | Malsi, Uttrakhand | 30.381955 | 78.053650 | HZ | 2016-17 |
| 33 | RJ-298 | Chikalwas, Rajasthan | 24.661862 | 73.681995 | PZ | 2016-17 |
| 34 | HP-366 | Joginder Nagar, Himachal Pradesh | 31.994232 | 76.793102 | HZ | 2016-17 |
| 35 | HP-367 | Joginder Nagar, Himachal Pradesh | 31.993068 | 76.792579 | HZ | 2016-17 |
| 36 | UK-393 | Raipur, Uttrakhand | 30.301807 | 78.100567 | HZ | 2016-17 |
| 37 | HR-394 | Magho Majri, Haryana | 29.815784 | 76.366425 | PZ | 2016-17 |
| 38 | UP-395 | Balli Pura, Uttar Prdaesh | 25.953318 | 83.557124 | PZ | 2016-17 |
| 39 | HP-397 | Banuri, Himachal Pradesh | 32.105405 | 76.563888 | HZ | 2016-17 |
| 40 | HP-399 | Chopati, Himachal Pradesh | 32.124373 | 76.537662 | HZ | 2016-17 |
| 41 | HP-400 | Nachhar, Himachal Pradesh | 32.142496 | 76.540600 | HZ | 2016-17 |
| 42 | HP-28 | Shimla, Himachal Pradesh | 31.086660 | 77.201275 | HZ | 2016-17 |
| 43 | HP-29 | Himachal Pradesh | 31.090325 | 77.146416 | HZ | 2016-17 |
| 44 | HP-34 | Una, Himachal Pradesh | 31.372113 | 76.275785 | HZ | 2016-17 |
| 45 | UK-44 | Aneki Hetmapur, Uttrakhand | 29.961059 | 78.039727 | HZ | 2016-17 |
| 46 | UK-51 | Manoharpur, Uttrakhand | 29.921498 | 78.030298 | HZ | 2016-17 |
| 47 | UK-53 | Salempur Mahdood, Uttrakhand | 29.943641 | 78.042956 | HZ | 2016-17 |
| 48 | UK-71 | Narendranagar, Uttrakhand | 30.308909 | 78.375537 | HZ | 2016-17 |
| 49 | UK-74 | Chamani, Uttrakhand | 30.328686 | 78.434675 | HZ | 2016-17 |
| 50 | HP-85 | Una, Himachal Pradesh | 31.546909 | 76.179597 | HZ | 2016-17 |
| 51 | UP-121 | Gaddou Pur, Uttar Pradesh | 26.761485 | 82.112990 | PZ | 2016-17 |
| 52 | RJ-130 | Benarwith Daulatpura, Rajasthan | 27.013230 | 75.732632 | PZ | 2016-17 |
| 53 | UP-135 | Saraiya, Uttar Pradesh | 26.963503 | 80.912122 | PZ | 2016-17 |
| 54 | UP-143 | Gomti Nagar, Uttar Pradesh | 26.826955 | 80.981990 | PZ | 2016-17 |
| 55 | UP-13 | Kalyanpur, Uttar Pradesh | 26.515911 | 80.263518 | PZ | 2016-17 |
| 56 | HP-18 | Shimla, Himachal Pradesh | 31.090325 | 77.146416 | HZ | 2016-17 |
| 57 | PB-14 | Ludhiana, Punjab | 30.933560 | 75.782741 | PZ | 2016-17 |
| 58 | HR-31 | Matehri Jattan, Haryana | 30.340846 | 76.786906 | PZ | 2016-17 |
| 59 | UP-32 | Chakeri Ward, Uttar Pradesh | 26.397339 | 80.455172 | PZ | 2016-17 |

PZ: Plain zone; HZ: Hill zone

| **Table S3:**  Comparative analysis of repeat motifs retrieved from the whole genome of *U. hordei* Uh364 and *U. hordei* Uh4857-4 | | | | | | | | | | | | |
| --- | --- | --- | --- | --- | --- | --- | --- | --- | --- | --- | --- | --- |
| Criteria | Mono | | Di | | Tri | | Tetra | | Penta | | Hexa | |
|  | Uh364 | Uh4857-4 | Uh364 | Uh4857-4 | Uh364 | Uh4857-4 | Uh364 | Uh4857-4 | Uh364 | Uh4857-4 | Uh364 | Uh4857-4 |
| Number | 12541 | 16332 | 1312 | 1564 | 20600 | 29462 | 1138 | 1428 | 22803 | 29336 | 14471 | 20491 |
| Length | 96707 | 130953 | 25296 | 29290 | 209412 | 300087 | 16028 | 19712 | 232605 | 299265 | 186492 | 267246 |
| Percent | 17.21 | 16.56 | 1.8 | 1.59 | 28.27 | 29.88 | 1.56 | 1.45 | 31.29 | 29.75 | 19.86 | 20.78 |
| Average Length | 7.71 | 8.02 | 19.28 | 18.73 | 10.17 | 10.19 | 14.08 | 13.8 | 10.2 | 10.2 | 12.89 | 13.04 |
| Relative Abundance | 622.91 | 606.2 | 65.17 | 58.05 | 1023.21 | 1093.56 | 56.52 | 53 | 1132.63 | 1088.88 | 718.78 | 760.58 |
| Relative Density | 4803.46 | 4860.66 | 1256.46 | 1087.17 | 10401.55 | 11138.5 | 796.12 | 731.66 | 11553.56 | 11107.99 | 9263.11 | 9919.52 |
| Class I SSR | 331 | 609 | 462 | 518 | 542 | 632 | 120 | 134 | 170 | 206 | 424 | 585 |
| Class II SSR | 1366 | 1528 | 850 | 1046 | 3176 | 6043 | 1018 | 1294 | 22633 | 29130 | 14047 | 19906 |
| Longest SSR | A_50_ | T_71_ | CT_86_ | TG_40_ | TGT_43_ | CAA_73_ | AAGC_17_ ATTT_17_ | ATAA_17_, TGCT_17_ | CTTTT_13_ | AAATC_39_ | CCCTCG_20_ | CCCTAA_37_ |
| Class I SSR = Length ≥ 20; Class II SSR= Length 10 ≤ Length < 20 | | | | | | | | | | | | |

| **Table S4:**  The Overall Ewens-Watterson Test for Neutrality of SSR markers calculated by using 1000 simulated samples | | | | | | | |
| --- | --- | --- | --- | --- | --- | --- | --- |
| **Locus** | **Obs. Frequency** | **Min Frequency** | **Max Frequency** | **Mean*** | **SE*** | **L95*** | **U95*** |
| UHB3 | 0.5001 | 0.3333 | 0.9667 | 0.6826 | 0.0332 | 0.373 | 0.9665 |
| UHB4 | 0.7564 | 0.3333 | 0.9667 | 0.6896 | 0.0334 | 0.371 | 0.9665 |
| UHB5 | 0.6334 | 0.3333 | 0.9667 | 0.6701 | 0.0327 | 0.376 | 0.9665 |
| UHB6 | 0.3538 | 0.3333 | 0.9667 | 0.6796 | 0.0342 | 0.3713 | 0.9665 |
| UHB17 | 0.5484 | 0.3333 | 0.9667 | 0.6719 | 0.0323 | 0.3799 | 0.9665 |
| UHB20 | 0.4506 | 0.3333 | 0.9667 | 0.6804 | 0.0342 | 0.3703 | 0.9665 |
| UHB21 | 0.6099 | 0.3333 | 0.9667 | 0.6696 | 0.034 | 0.3638 | 0.9665 |
| UHB25 | 0.3473 | 0.3333 | 0.9667 | 0.6828 | 0.0336 | 0.3709 | 0.9665 |
| UHB26 | 0.3939 | 0.3333 | 0.9667 | 0.6737 | 0.0338 | 0.3709 | 0.9665 |
| UHB27 | 0.5685 | 0.3333 | 0.9667 | 0.6746 | 0.0317 | 0.3673 | 0.9502 |
| UHB28 | 0.403 | 0.3333 | 0.9667 | 0.6806 | 0.0344 | 0.3806 | 0.9665 |
| UHB30 | 0.4599 | 0.3333 | 0.9667 | 0.6875 | 0.0328 | 0.3657 | 0.9665 |
| UHB31 | 0.4484 | 0.3333 | 0.9667 | 0.6698 | 0.0315 | 0.3623 | 0.9665 |
| UHB32 | 0.6553 | 0.3333 | 0.9667 | 0.681 | 0.0328 | 0.3749 | 0.9665 |
| UHB36 | 0.5702 | 0.3333 | 0.9667 | 0.6765 | 0.0325 | 0.388 | 0.9665 |

| **Table S5:**  Diversity indices of 15 microsatellite loci used in the study | | | | | | | |
| --- | --- | --- | --- | --- | --- | --- | --- |
| **Primer** | **Na** | **N_e_** | **I** | **Fst** | **Nm** | **He** | **PIC (%)** |
| UHB3 | 2 | 1.999 | 0.784 | 0.2669 | 0.6866 | 0.0997 | 0.0948 |
| UHB4 | 2 | 1.322 | 0.491 | 0.1158 | 1.9085 | 0.1349 | 0.1258 |
| UHB5 | 2 | 1.579 | 0.674 | 0.0987 | 2.2818 | 0.1769 | 0.1612 |
| UHB6 | 2 | 2.827 | 1.069 | 0.1231 | 1.7807 | 0.4767 | 0.3631 |
| UHB17 | 2 | 1.824 | 0.799 | 0.2413 | 0.7858 | 0.2688 | 0.2327 |
| UHB20 | 2 | 2.219 | 0.910 | 0.1651 | 1.2640 | 0.4842 | 0.367 |
| UHB21 | 2 | 1.639 | 0.712 | 0.2170 | 0.9022 | 0.233 | 0.2058 |
| UHB25 | 2 | 2.879 | 1.076 | 0.1316 | 1.6495 | 0.4904 | 0.3702 |
| UHB26 | 2 | 2.539 | 1.010 | 0.4262 | 0.3365 | 0.3866 | 0.3119 |
| UHB27 | 2 | 1.759 | 0.770 | 0.4029 | 0.3705 | 0.2408 | 0.2118 |
| UHB28 | 2 | 2.481 | 0.990 | 0.2474 | 0.7606 | 0.4228 | 0.3334 |
| UHB30 | 2 | 2.174 | 0.908 | 0.3248 | 0.5197 | 0.2726 | 0.2354 |
| UHB31 | 2 | 2.230 | 0.939 | 0.1831 | 1.1153 | 0.375 | 0.3047 |
| UHB32 | 2 | 1.526 | 0.646 | 0.2727 | 0.6667 | 0.2008 | 0.1806 |
| UHB36 | 2 | 1.754 | 0.764 | 0.2710 | 0.6725 | 0.2149 | 0.1918 |
| Na: Number of alleles; I=; Ne= number of effective alleles; He = expected heterozygosity; and PIC = polymorphism information content | | | | | | | |

| **Table S6:**  Analysis of molecular variance (AMOVA) for UH isolates from Northern parts of India | | | | | | | |
| --- | --- | --- | --- | --- | --- | --- | --- |
| Source of variation | df | Sum of squares | MS | Variance components | Variation (%) |  | P-value |
| Among population | 1 | 41.247 | 41.247 | 0.740 | 13 |  | < 0.001 |
| Within population | 59 | 280.000 | 4.746 | 4.746 | 87 |  | < 0.001 |
| Total | 117 | 391.237 |  | 5.485 | 100 |  |  |
| Df: degree of freedon; Ms: means | | | | | | | |

| **Table S7:**  F-statistics analysis and pair-wise comparisons of genetic distance (Fst ), genetic flow (Nm) and Nei’s unbiased genetic identity for UH isolates collected from the barley fields | | | | | | |
| --- | --- | --- | --- | --- | --- | --- |
| Population 1 | Population 2 | F_st_ | F_it_ | F_is_ | N_m_ | Nei’s unbiased genetic identity |
| HZ(N=38) | PZ (N=21) | 0.248 | -0.274 | -0.589 | 1.009 | 0.198 |
| F_st_ = AP/(WI + AI + AP) = AP/TOT; F_is_, AI/(WI + AI); F_it_, (AI + AP)/(WI + AI + AP) = (AI+ AP)/TOT; N_m_ = [(1/F_st_)-1]/4]; where Est. Var. Among zones, AI=Estimated variance among isolates; WI = Estimated variance within isolates | | | | | | |
